# Supplementary material for: Pretreatment [18F]FDG PET/CT Prognostic Factors in Patients with Squamous Cell Cervical Carcinoma FIGO IIIC1
Source: Diagnostics (Basel). 2021 Apr 16;11(4):714. doi: 10.3390/diagnostics11040714 (PMC8073234; doi:10.3390/diagnostics11040714)
Supplement: Supplementary file 1 [file diagnostics-11-00714-s001.zip › diagnostics-1130222-supplementary/FIGO 2018 staging.pdf]

FIGO 2018 staging

In 2018 FIGO staging has been revised by the FIGO Gynecologic Oncology Committee to allow imaging and pathological findings, where available, to supplement clinical findings with respect to tumor size and extent, in all stages. The involvement of vascular/lymphatic spaces does not change the staging. The lateral extent of the lesion is no longer considered. Adding notation of r (imaging) and p (pathology) to indicate the findings that are used to allocate the case to Stage IIIC.

Stage Description
I The carcinoma is strictly confined to the cervix (extension to the uterine corpus should be disregarded)
IA Invasive carcinoma that can be diagnosed only by microscopy, with maximum depth of invasion <5 mma
IA1 Measured stromal invasion <3 mm in depth
IA2 Measured stromal invasion ≥3 mm and <5 mm in depth
IB Invasive carcinoma with measured deepest invasion ≥5 mm (greater than Stage IA), lesion limited to the cervix
IB1 Invasive carcinoma ≥5 mm depth of stromal invasion, and <2 cm in greatest dimension
IB2 Invasive carcinoma ≥2 cm and <4 cm in greatest dimension
IB3 Invasive carcinoma ≥4 cm in greatest dimension
II The carcinoma invades beyond the uterus, but has not extended onto the lower third of the vagina or to the pelvic wall
IIA Involvement limited to the upper two-thirds of the vagina without parametrial involvement
IIA1 Invasive carcinoma <4 cm in greatest dimension
IIA2 Invasive carcinoma ≥4 cm in greatest dimension
