# Supplementary material for: Pretreatment [18F]FDG PET/CT Prognostic Factors in Patients with Squamous Cell Cervical Carcinoma FIGO IIIC1
Source: Diagnostics (Basel). 2021 Apr 16;11(4):714. doi: 10.3390/diagnostics11040714 (PMC8073234; doi:10.3390/diagnostics11040714)
Supplement: Supplementary file 1 [file diagnostics-11-00714-s001.zip › diagnostics-1130222-supplementary/Table 2 FGD PET CT new DFS no (1).pdf]

Table2b FDG PET CT parameters of patients with reccurence

	n	Average	Median	Min	Max	SD
Activity of FDG (mCi)	25	8,344	8,300	5,6000	12,70	1,722
Glucose level (mg%)	25	90,080	87,000	56,0000	137,00	18,312
SUVmax	25	13,536	11,680	6,6500	26,86	5,955
SUVmean	25	7,781	6,380	3,7700	14,91	3,261
TumorSUV	25	5307,085	3929,240	652,8600	26057,90	5165,840
TLG	25	330,058	245,640	26,0300	1667,71	336,277
MTV	25	39,190	36,910	7,0000	115,25	23,316
SUVtotal	25	23,309	20,110	11,0500	45,10	9,050
TLGtotal	25	351,662	265,690	45,5600	1672,91	329,947
MTVtotal	25	42,764	38,960	8,0600	116,56	23,164
SUVLN	25	9,773	7,530	2,9100	25,58	6,232
TLGLN	25	21,604	8,040	1,0600	242,80	47,475
MTVLN	25	3,574	2,570	0,4200	10,82	2,810
AUC-CSH	25	0,579	0,576	0,5200	0,65	0,034
Heterogenity	25	0,269	0,272	0,2120	0,31	0,022

MTV - metabolic target volume (MTV) of the cervical tumor, MTVLN - MTV obtained from all the metastatic pelvic lymph nodes (PLN), MTVtotal - the sum of MTV of tumor and MTVLN

SD - standard deviation, SUVLN - maximum standardized uptake value of PLN, SUVmax - maximum standardized uptake value of the cervical tumor, SUVmean - mean of standard uptake value of the cervical tumor, SUVtotal the sum of SUVmax of a tumor and SUVmax obtained from all the metastatic PLN, TLG - total lesion glycolysis of the cervical tumor, TLGLN- total lesion glycolysis obtained from all the metastatic PLN, TLGtotal- the sum of TLG of tumor and TLG obtained from all the metastatic PLN, TumorSUV is the sum of all SUV values within the tumor
