# Supplementary material for: Pretreatment [18F]FDG PET/CT Prognostic Factors in Patients with Squamous Cell Cervical Carcinoma FIGO IIIC1
Source: Diagnostics (Basel). 2021 Apr 16;11(4):714. doi: 10.3390/diagnostics11040714 (PMC8073234; doi:10.3390/diagnostics11040714)
Supplement: Supplementary file 1 [file diagnostics-11-00714-s001.zip › diagnostics-1130222-supplementary/Table 2 FGD PET CT new DFS Yes (1).pdf]

Table 2a FDG PET CT parameters of patients free of disease

	n	Average	Median	Min	Max	SD
Activity of FDG (mCi)	68	9,166	8,750	4,3000	13,10	1,716
Glucose level (mg%)	68	91,221	88,000	60,0000	160,00	17,258
SUVmax	68	14,353	13,275	4,5600	32,61	5,366
SUVmean	68	8,353	7,695	2,5800	17,93	3,171
TumorSUV	68	4992,336	3847,545	925,7000	20205,41	3871,716
TLG	68	319,520	246,260	59,2400	1293,15	247,797
MTV	68	36,275	33,610	9,4800	87,78	18,460
SUVtotal	68	23,274	21,930	10,6300	44,98	8,836
TLGtotal	68	355,113	255,975	65,2100	1769,20	303,581
MTVtotal	68	39,550	38,590	11,0100	90,00	19,227
SUVLN	68	8,921	7,440	1,9400	26,47	5,546
TLGLN	68	35,593	7,310	0,1000	1667,97	201,298
MTVLN	68	3,275	2,475	0,0500	12,97	2,790
AUC-CSH	68	0,582	0,579	0,5170	0,71	0,039
Heterogenity	68	0,268	0,271	0,1710	0,32	0,026

MTV - metabolic target volume (MTV) of the cervical tumor, MTVLN - MTV obtained from all the metastatic pelvic lymph nodes (PLN), MTVtotal - the sum of MTV of tumor and MTVLN

SD - standard deviation, SUVLN - maximum standardized uptake value of PLN, SUVmax - maximum standardized uptake value of the cervical tumor, SUVmean - mean of standard uptake value of the cervical tumor, SUVtotal the sum of SUVmax of a tumor and SUVmax obtained from all the metastatic PLN, TLG - total lesion glycolysis of the cervical tumor, TLGLN- total lesion glycolysis obtained from all the metastatic PLN, TLGtotal- the sum of TLG of tumor and TLG obtained from all the metastatic PLN, TumorSUV is the sum of all SUV values within the tumor
